# The complete chloroplast genome sequence of a cultivar of *Chrysanthemum*, *Chrysanthemum morifolium* var. ‘Jinsihuang’ (Asteraceae)

**DOI:** 10.1080/23802359.2026.2621429

**Published:** 2026-02-01

**Authors:** Wenying Meng, Qimao Wang, Yonghao Wang, Feng Wen, Xiaozhu Wu

**Affiliations:** ^a^ School of Life Sciences, Anhui University, Hefei, China; ^b^ School of Biological Science and Food Engineering, Anhui Chuju Planting and Deep Processing Engineering Research Center, Chuzhou University, Chuzhou, China; ^c^ School of Resources Environmental Sciences, Jiujiang University, Jiujiang, China

**Keywords:** Chloroplast genome, *Chrysanthemum morifolium* var. ‘Jinsihuang’, phylogenetic analyses

## Abstract

*Chrysanthemum morifolium* var. ‘Jinsihuang’, an ornamental chrysanthemum with notable medicinal and edible value, had its complete chloroplast (cp) genome sequenced, assembled, and annotated in this study. The cp genome is 151,060 bp long, exhibiting a typical quadripartite structure: an 82,858 bp large single-copy (LSC) region, an 18,294 bp small single-copy (SSC) region, and two inverted repeat (IR) regions (24,954 bp each). Phylogenetic analysis revealed that it is most closely related to *Chrysanthemum* × *morifolium*. This study provides valuable genomic resources for understanding its phylogenetic relationships and genetic diversity, contributing to future research on its evolution and conservation.

## 1. Introduction

**Figure 1.**
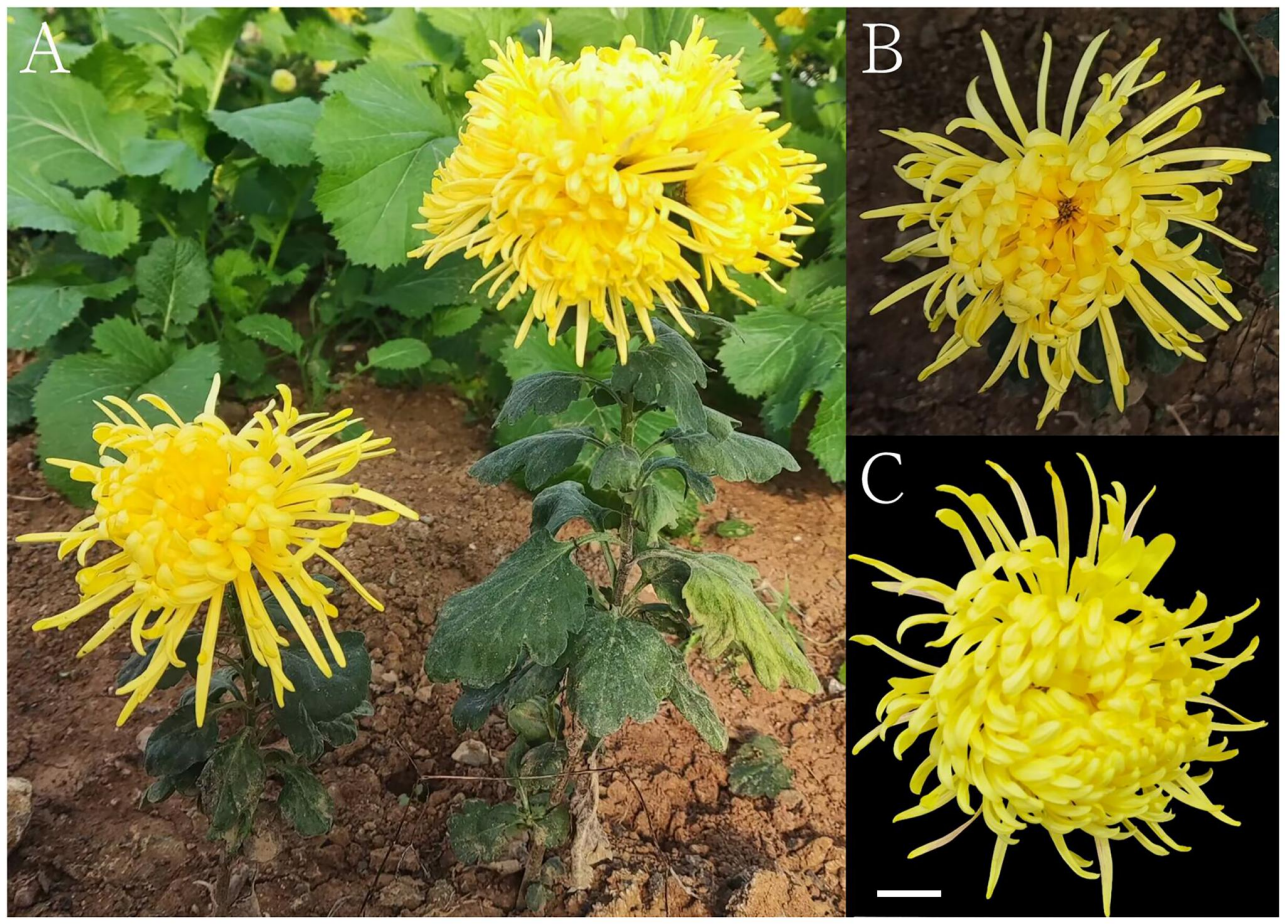
The morphological characteristics of *C. morifolium* var. ‘Jinsihuang’. (A) Planting site and blooming whole plant. (B) Blooming flowers. (C) Size of the flowers. The scale bar represents 2 cm. Photographs were taken by Wenying Meng at Chuzhou University (32°41′03ʺN, 118°36′51ʺE), and a voucher specimen (CZU2023001) is deposited in its Herbarium (CZU), Chuzhou, Anhui, China.

*Chrysanthemum morifolium* var. ‘Jinsihuang’ (Asteraceae), a perennial herb, is a hybrid variety derived from traditional medicinal and ornamental chrysanthemums (Figure 1). In recent years, it has gained wide popularity among consumers and been exported globally due to its dual value for ornamental and edible purposes. Rich in flavonoids, this cultivar exhibits diverse biological activities (antioxidant, anti-inflammatory, antibacterial, and antiviral) (Liu et al. 2001; Kim and Kim 2011) and has traditionally been used in Chinese medicine to treat ailments including headaches, eye fatigue, hypertension, and various cardiovascular diseases (Hadizadeh et al. 2022). However, molecular-level studies on *C. morifolium* var. ‘Jinsihuang’ remain limited, and its genetic resources are scarce. In this study, we successfully assembled its complete chloroplast (cp) genome and elucidated its phylogenetic position within the *Chrysanthemum* genus. By reporting the first complete plastome of this cultivar, we aim to provide foundational molecular data that will facilitate future comparative genomic, genetic, and taxonomic studies within both the *Chrysanthemum* genus and the Asteraceae family.

## 2. Materials and methods

Fresh leaves of *C. morifolium* var. ‘Jinsihuang’ were collected from the greenhouse of the Planting and Deep Processing Engineering Research Center, Chuzhou University, Anhui Province, China (32°41′03ʺN, 118°36′51ʺE). A voucher specimen (CZU2023001) is deposited in the Herbarium of the School of Biological and Food Engineering, Chuzhou University under the custody of Dr. Feng Wen (email: wenfeng332@126.com). Genomic DNA was extracted from fresh leaves with a modified CTAB method (Doyle and Doyle 1987). To mitigate polyphenol and flavonoid interference, PVP-40 concentration to 3% and β-mercaptoethanol to 2% were added. Subsequently, sequencing libraries were constructed following the guidelines provided by the MGIEasy Universal DNA Library Preparation Kit. Afterwards, the 150 bp fragments were sequenced from both ends by utilizing the Illumina HiSeq 2500 platform at Genepioneer Biotechnologies Inc. (Nanjing, China). The raw sequencing data were filtered and trimmed with the help of fastp (version 0.23.4) under its default settings (Chen et al. 2018). Altogether, 19.24 million high-quality clean reads were generated, resulting in approximately 5.77 Gb of data. The complete cp genome was assembled using GetOrganelle (version 1.7.7.0). Only top 11,408,361 pair of reads were used for analysis, and 932,053 pair of reads (approximately 8.17% of total used clean reads) were used to assemble the cp genome. The subsequent annotation performed through the GeSeq web-based annotation platform for comprehensive genomic characterization (Tillich et al. 2017; Jin et al. 2020). Annotation accuracy was verified using BLAST (https://blast.ncbi.nlm.nih.gov/Blast.cgi) and manual curation. A circular genome map was generated with OGDRAW (Greiner et al. 2019) using *Arabidopsis thaliana* (NC_000932) as a reference. The start and stop codons were manually corrected based on BLAST results, while cis-spliced and trans-spliced gene profiles were constructed by utilizing CPGview (http://www.1kmpg.cn/cpgview/) (Liu et al. 2023). The boundaries of the LSC, SSC, and IR regions were compared using CPJSdraw (Li et al. 2023). To evaluate the phylogenetic relationships, cp genomes of 17 *Chrysanthemum* species were retrieved from the NCBI and aligned using ClustalW (Thompson et al. 1994). All sequences from the alignment were used for tree construction without trimming. A maximum-likelihood (ML) tree was constructed with MEGA 11 using the GTR + G model (selected as best-fit via AIC) and 1000 bootstrap replicates, with *Helianthus annuus* as the outgroup (Tamura et al. 2021).

## 3. Results

**Figure 2.**
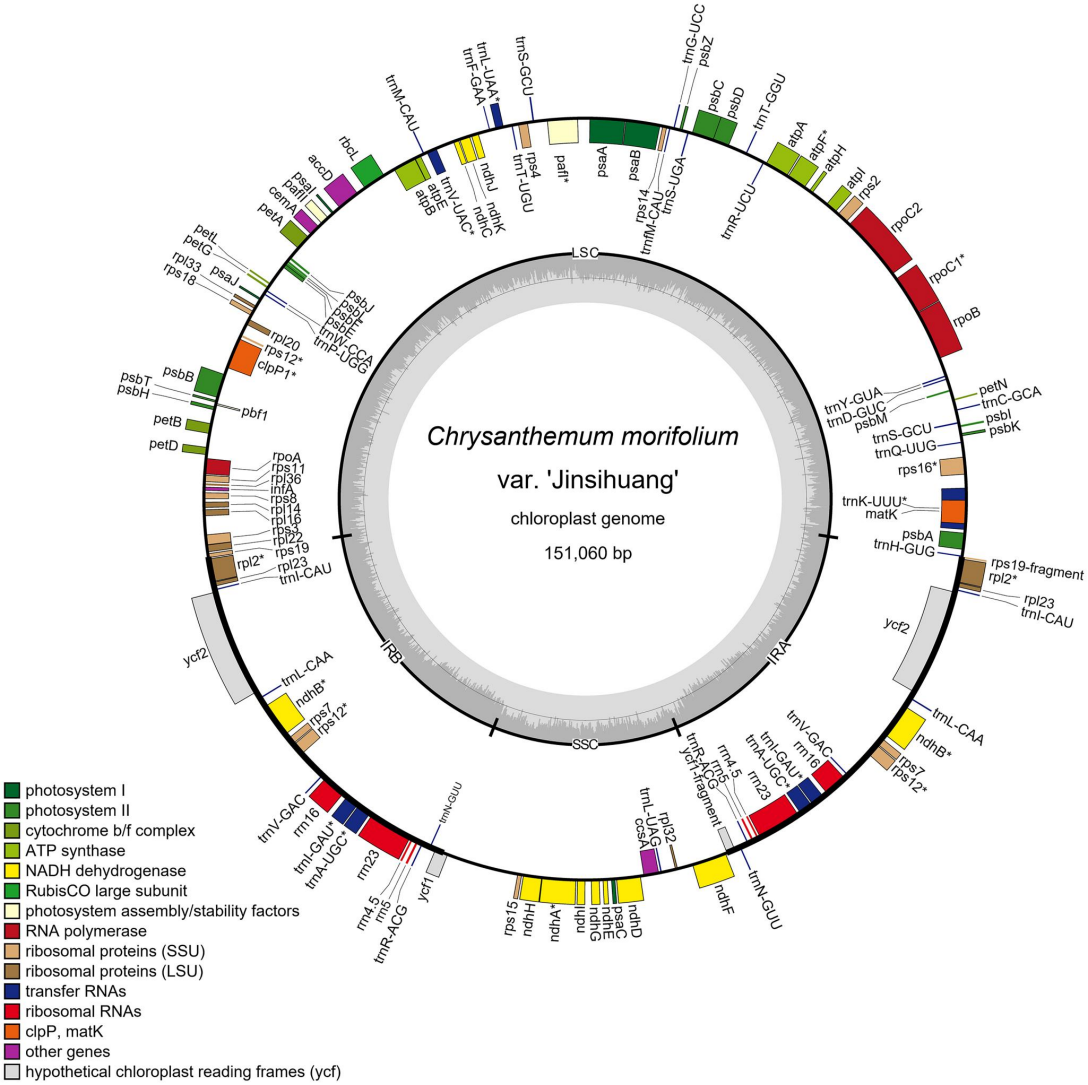
Chloroplast genome mapping of *C. morifolium* var. ‘Jinsihuang’. The inner circle is a pair of IR regions, LSC and SSC regions. Forward-coding genes are located on the outer side of the circle, while reverse-coding genes are located on the inner side of the circle. The inner gray area circles indicate the GC content. At the bottom, genes with different functional groups are indicated by various colors.

The complete cp genome of *C. morifolium* var. ‘Jinsihuang’ was 151,060 bp in length, with mean, maximum, and minimum sequencing depths of 818.64×, 1051×, and 183×, respectively (Supplementary Figure 1). The genome structure comprises a large single-copy (LSC) region with a length of 82,858 bp, a small single-copy (SSC) region measuring 18,294 bp, and two inverted repeat (IR) regions, each being 24,954 bp long, displaying a typical quadripartite structure found in many angiosperms (Figure 2) (Wicke et al. 2011). The annotated cp genome sequence has been submitted to GenBank with the accession number PQ759883.

The overall GC content of the cp genome amounts to 37.5%, with region-specific values being 35.5% in the LSC region, 30.8% in the SSC region, and 43.1% in the IR regions (Supplementary Table 1). The genome contains a total of 130 predicted gene loci. The annotation comprises 87 protein-coding genes (PCGs), 35 tRNA genes, and eight rRNA genes, with two additional pseudogenes identified. In total, 17 of these genes are duplicated, including *ndhB*, *rpl2*, *rpl23*, *rps7*, *rps12*, *rrn4.5*, *rrn5*, *rrn16*, *rrn23*, *trnA-UGC*, *trnI-CAU*, *trnI-GAU*, *trnL-CAA*, *trnN-GUU*, *trnR-ACG*, *trnS-GCU*, and *trnV-GAC* (Supplementary Table 2). Six PCGs (*atpF*, *rpl2*, *rpoC1*, *rps16*, *ndhA*, and *ndhB*) contain a single intron, while two PCGs (*pafI*, *clpP*) have two introns. Notably, the *ndhB* and *rpl2* genes each feature a single duplication (Supplementary Figure 2), while the *rps12* gene undergoes trans-splicing and comprises three distinct exons (Supplementary Figure 3). Although, the cp genome of *C. morifolium* var. ‘Jinsihuang’ shows significant similarities to other species, differences exist at the junctions of the LSC-IRa (LA) and SSC-IRb (SB) regions compared with specified cultivars and other congeneric *Chrysanthemum* species (Supplementary Figure 4). These structural similarities and differences may indicate the evolutionary connections and relationships among them.

**Figure 3.**
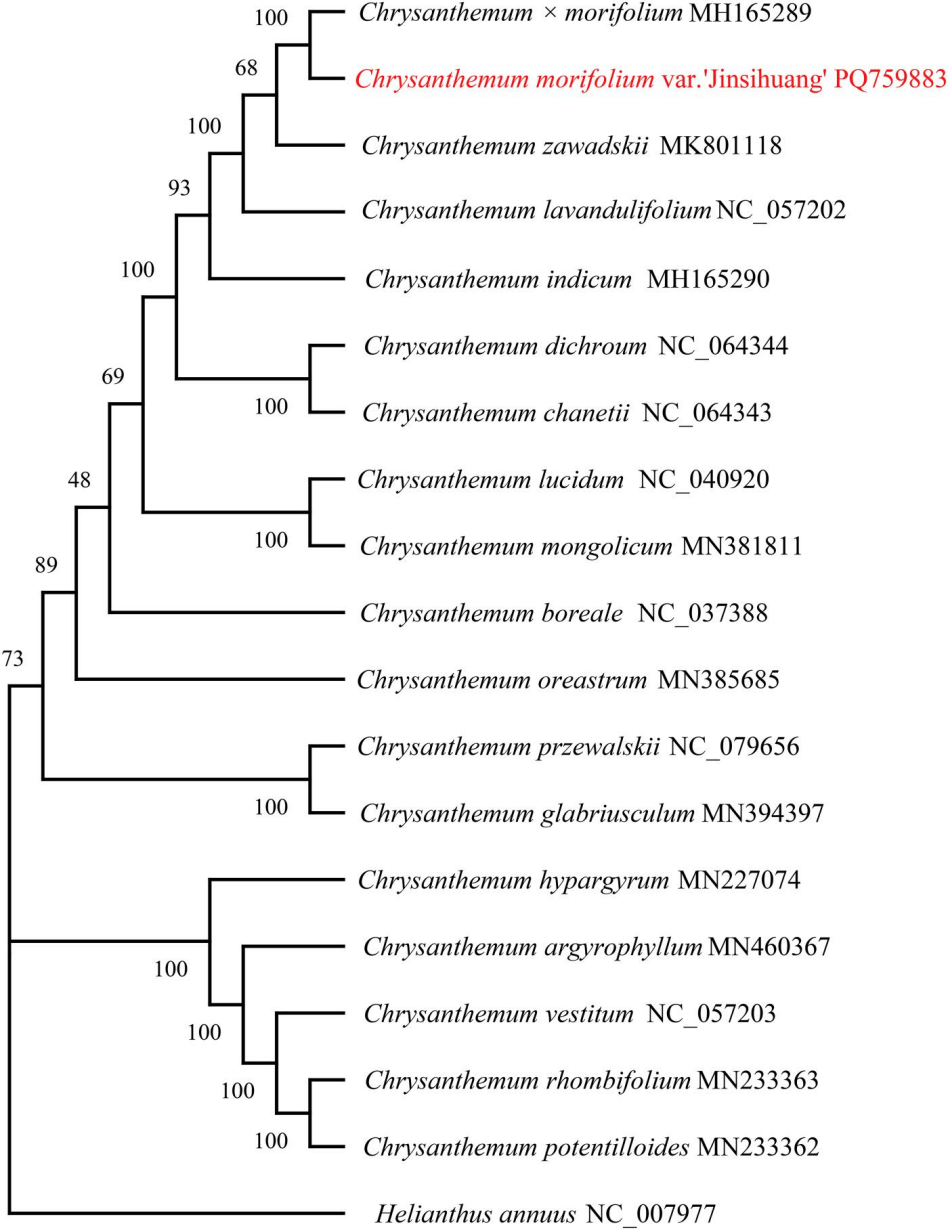
Construction of the ML tree based on the chloroplast genomes of 19 species using MEGA 11 and 1000 bootstrap replicates; bootstrap support values are labeled at each branch node. The following sequences were used: MH165289 (Ma et al. 2020), PQ759883, NC_057203, NC_057202 (Ma et al. 2020), MH165290 (Xia et al. 2016), NC_079656, NC_064344, NC_064343, NC_037388 (Won et al. 2018), NC_040920 (Kim et al. 2018), MK801118 (Baek et al. 2021), MN394397, MN385685, MN381811, MN460367, MN233363, MN233362, MN227074, and NC_007977.

The phylogenetic tree demonstrated that *C. morifolium* var. ‘Jinsihuang’ has the closest genetic relationship with *Chrysanthemum* × *morifolium* (Figure 3), accompanied by a high bootstrap support value of 100. This tree provides valuable insights into the phylogenetic relationships among *Chrysanthemum* species, highlighting genetic differentiation and establishing a solid foundation for further research into the evolutionary and genetic dynamics of these species.

## 4. Discussion and conclusions

Chloroplasts are vital for sustaining life on earth, and studying cp genomes offers invaluable insights into the interplay between cp DNA structure and species evolution (Daniell et al. 2016). In the present study, structural analysis of the *C. morifolium* var. ‘Jinsihuang’ cp genome revealed two key observations regarding IR regions: first, its IR region boundaries exhibit significant similarity to those of other *Chrysanthemum* species, reflecting the conserved nature of IR structures in maintaining cpDNA stability; second, distinct variations were detected at the LSC-IRa (LA) and SSC-IRb (SB) junctions, specifically a shorter *ycf1* gene and a truncated pseudogenized *rps19*, when compared with specified *C. morifolium* cultivars as well as other congeneric *Chrysanthemum* species (Supplementary Figure 4). These junction-specific differences are likely evolutionary signatures, as IR-SC boundaries are known to be dynamic hotspots for cpDNA rearrangement and pseudogenization (Cosner et al. 2004; Mehmood et al. 2021).

Beyond boundary features, the cp genome of ‘Jinsihuang’ encodes 130 genes, with 17 genes present in duplicate. Gene duplication in IR regions is thought to enhance cpDNA copy number stability and provide functional redundancy, which may buffer against deleterious mutations. Meanwhile, the identification of two pseudogenes in its cp genome provides a unique perspective on genomic evolutionary dynamics (Mehmood et al. 2021). Specifically, the *ycf1*-fragment pseudogene may influence the regulation of cp gene expression, potentially affecting cp development and function. Additionally, the *rps19*-fragment pseudogene could impact cp protein synthesis. The failure to produce functional ribosomal small subunit protein S19 may disrupt the normal assembly of cp ribosomes, highlighting the importance of these genetic elements in cp biology.

Phylogenetic analyses based on cp genome sequences reveal that *C. morifolium* var. ‘Jinsihuang’ is most closely related to *Chrysanthemum* × *morifolium*. This close genetic relationship suggests that these species may have faced similar evolutionary selection pressures, leading to shared genetic variations (Duan et al. 2024). Furthermore, the unique LA and SB junction differences between *C. morifolium* var. ‘Jinsihuang’ and other *Chrysanthemum* species offer essential clues for investigating the mechanisms underlying species differentiation within the genus—for instance, whether such structural variations contribute to phenotypic divergence or ecological adaptation.

In summary, our study not only enhances the genomic information of *C. morifolium* var. ‘Jinsihuang’, but also provides a foundation for understanding genetic diversity, evolution, and phylogenetic relationships within the *Chrysanthemum* genus.

## Supplementary Material

Supplementary Figures.docx

Supplementary Table.docx

Clean Version.docx

## Data Availability

The genome sequence data that support the findings of this study are openly available in GenBank of NCBI (https://www.ncbi.nlm.nih.gov/) under the accession number PQ759883. The associated BioProject, SRA, and Bio-Sample numbers are PRJNA1214688, SRR33442979, and SAMN46381781, respectively.
