# Supplementary material for: The complete chloroplast genome sequence of a cultivar of Chrysanthemum, Chrysanthemum morifolium var. ‘Jinsihuang’ (Asteraceae)
Source: Mitochondrial DNA B Resour. 2026 Feb 1;11(3):345–9. doi: 10.1080/23802359.2026.2621429 (PMC12865824; doi:10.1080/23802359.2026.2621429)
Supplement: Supplementary Figures.docx [file TMDN_A_2621429_SM6550.docx]

**Supplementary Figures**


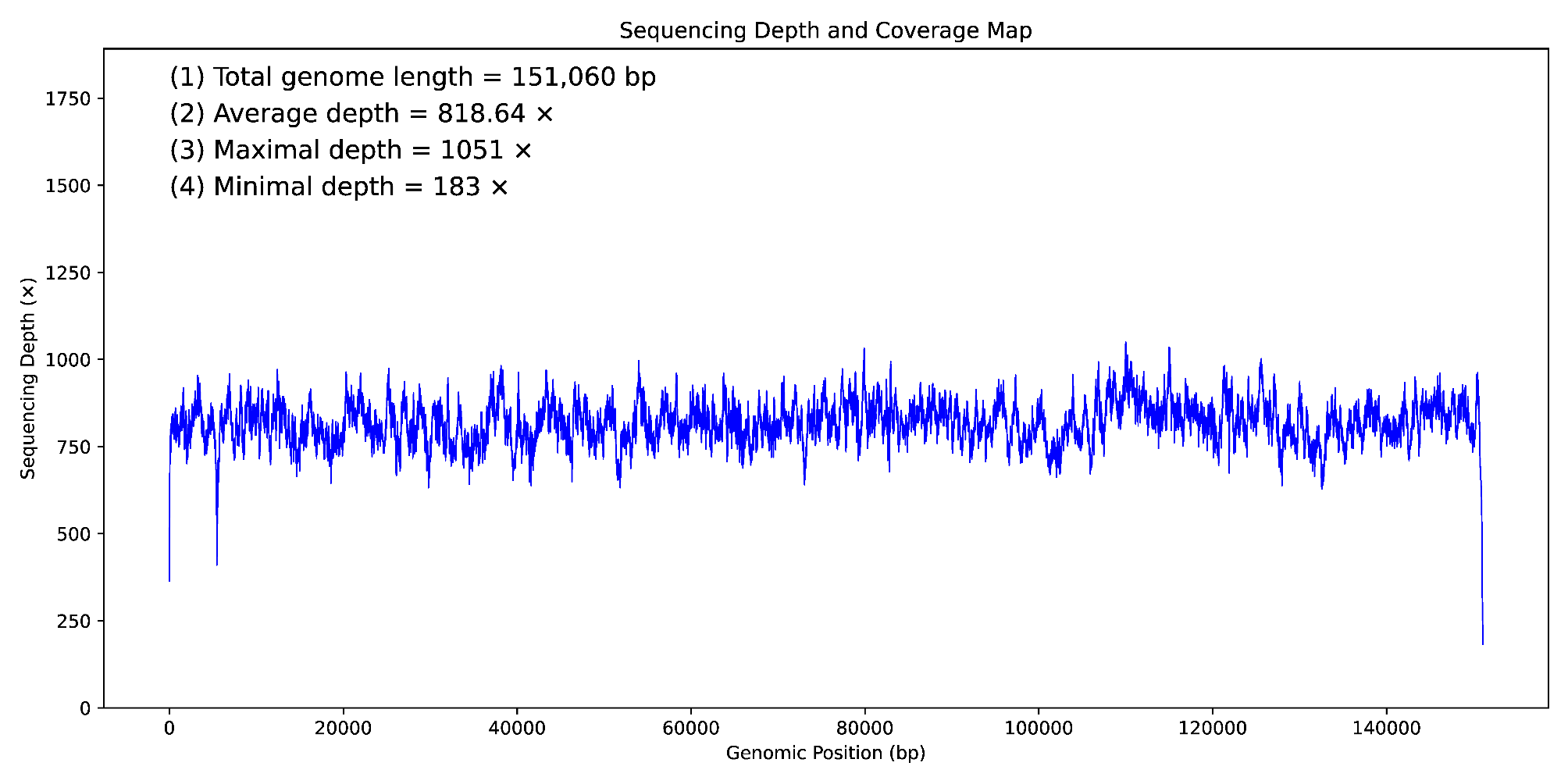


**Supplementary Figure 1.** Coverage depth distribution of the *C.morifolium* var. 'Jinsihuang' cp genome.


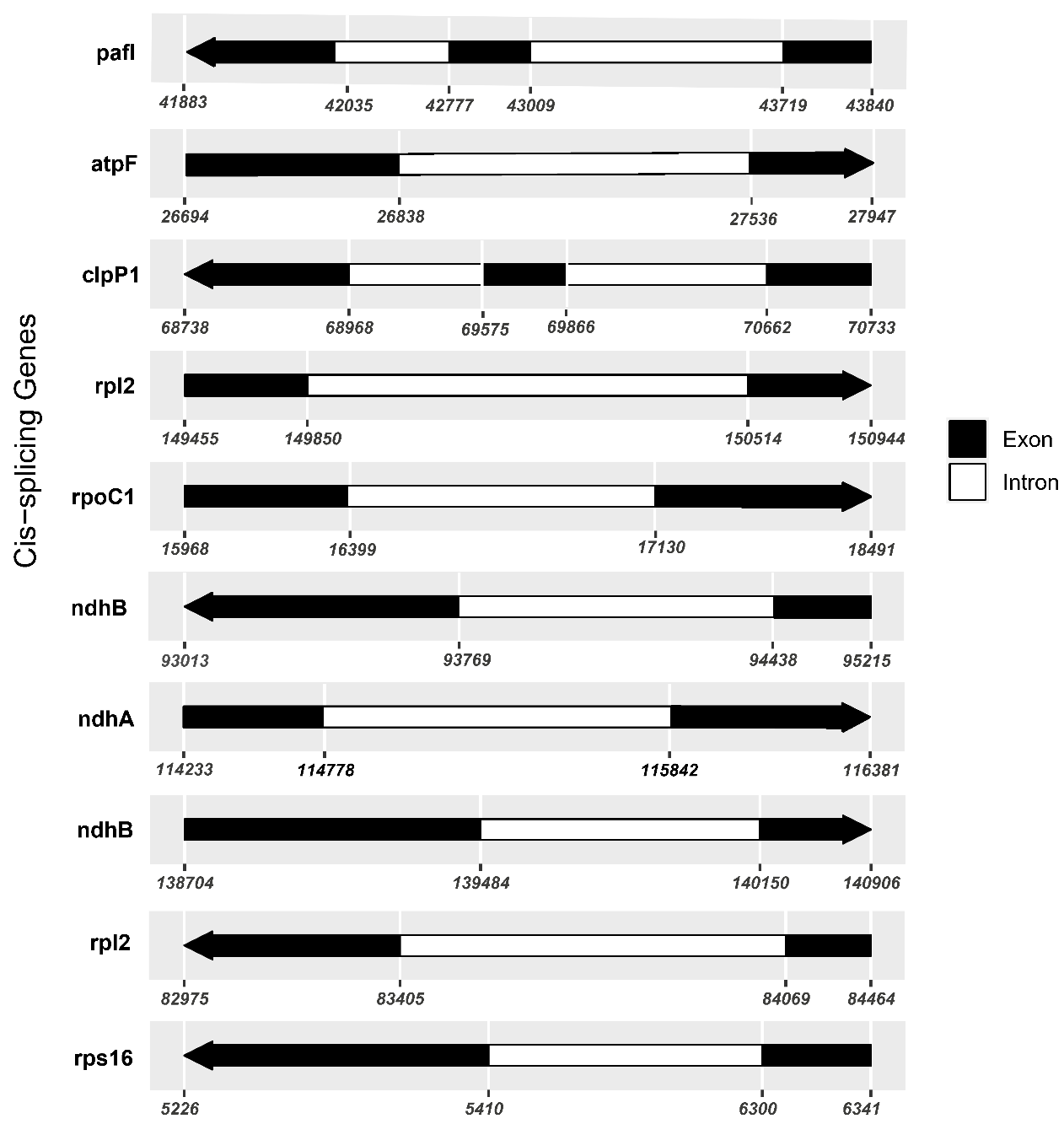


**Supplementary Figure 2.** Structure of cis-splicing genes in the *C.morifolium* var. 'Jinsihuang' cp genome.


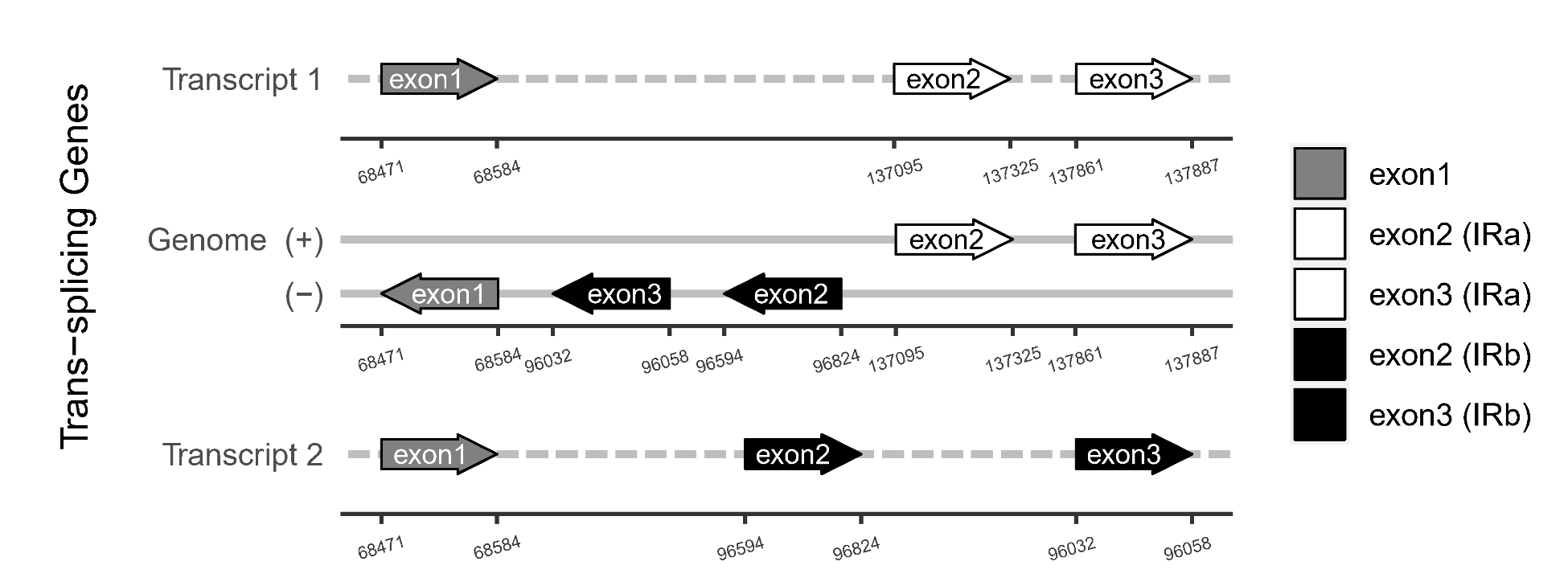


**Supplementary Figure 3.** Structure of trans-splicing genes in the *C.morifolium* var. 'Jinsihuang' cp genome.


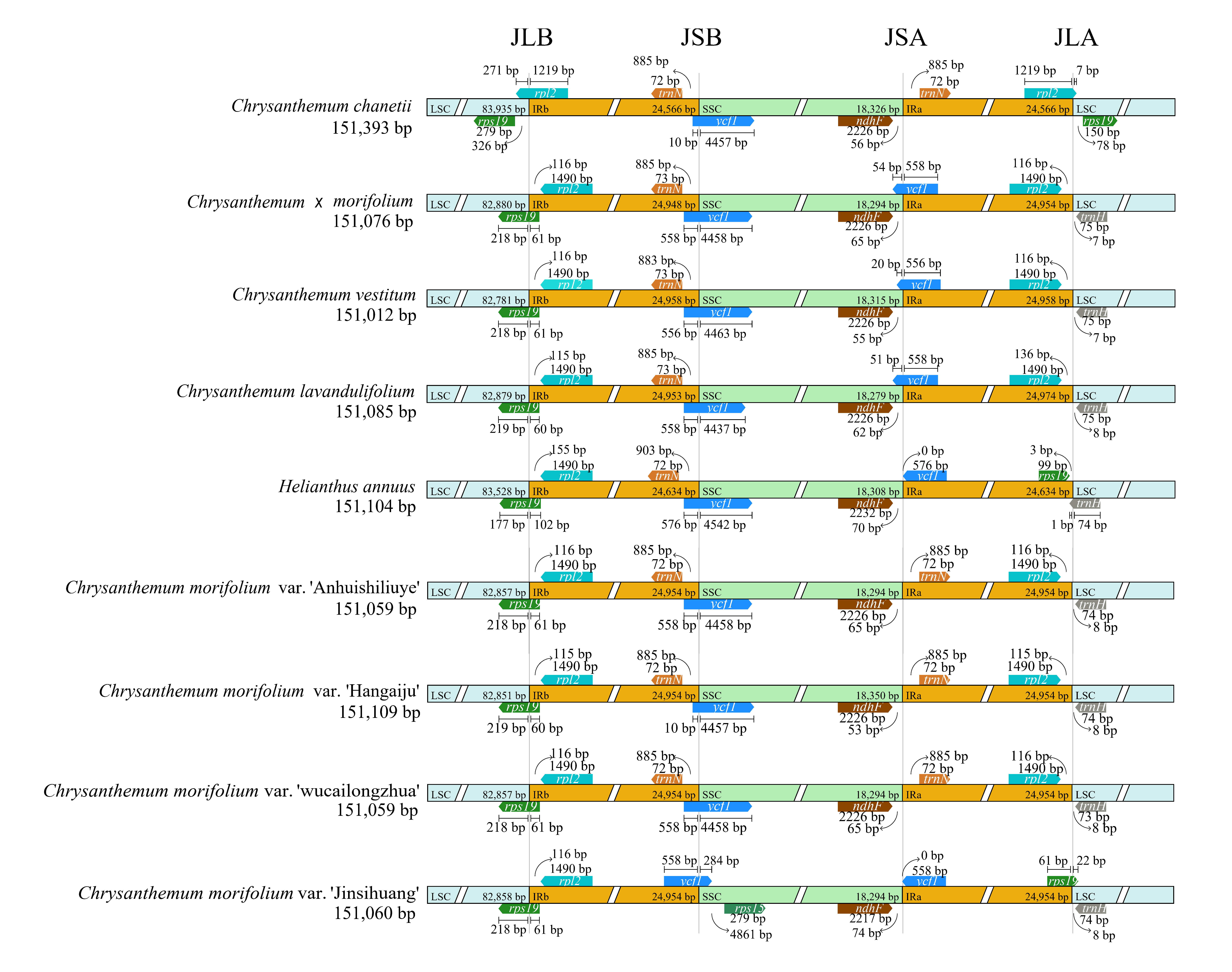


**Supplementary Figure 4.** Schematic diagram of IR boundaries (LSC-IRb (JLB), IRb-SSC (JSB), SSC-Ira (JSA), and IRa-LSC(JLA)) for 8 Chrysanthemum species (*C. chanetii*, C *× morifolium, C. vestitum, C. lavandulifolium, H. annuus, C. morifolium* var. *‘Anhuishiliuye’, C. morifolium* var. *'Hangbaiju', C. morifolium* var. *‘wucailongzhua’ and C. morifolium* var. 'Jinsihuang') and *H. annuus*.
