# Supplementary material for: The complete chloroplast genome sequence of a cultivar of Chrysanthemum, Chrysanthemum morifolium var. ‘Jinsihuang’ (Asteraceae)
Source: Mitochondrial DNA B Resour. 2026 Feb 1;11(3):345–9. doi: 10.1080/23802359.2026.2621429 (PMC12865824; doi:10.1080/23802359.2026.2621429)
Supplement: Supplementary Table.docx [file TMDN_A_2621429_SM6549.docx]

**Supplementary Table1.** Summary of *C. morifolium* var. *'*Jinsihuang' chloroplast genome

structure

| Region name | Start (bp) | End (bp) | Length (bp) | GC content (%) |
| --- | --- | --- | --- | --- |
| LSC | 1 | 82,858 | 82,858 | 35.50 |
| IRB | 82,859 | 107,812 | 24,954 | 43.10 |
| SSC | 107,813 | 126,106 | 18,294 | 30.80 |
| IRA | 126,107 | 151,060 | 24,954 | 43.10 |

**Supplementary Table2.** Gene composition in the chloroplast of *C. morifolium* var. 'Jinsihuang'

| Category for genes | Group of gene | Name of gene |
| --- | --- | --- |
| Photosynthesis | Subunits of ATP synthase | *atpA, atpB, atpE, atpF, atpH, atpI* |
|  | Subunits of photosystem II | *psbE, psbF* |
|  | Subunits of cytochrome b/f complex | *petA, petB, petD, petG, petL, petN* |
|  | Subunits of NADH-dehydrogenase | *ndhA*, ndhB*(x2), ndhC, ndhD, ndhE, ndhF, ndhG, ndhH, ndhI, ndhJ, ndhK* |
|  | Assembly/stability of photosystem I | *pafI*, pafII* |
|  | Subunits of photosystem I | *psaA, psaB, psaC, psaI, psaJ* |
|  | Subunits of photosystem II | *psbA, psbB, psbC, psbD, psbH, psbI, psbJ, psbK, psbL, psbM, psbT, psbZ* |
|  | Cytochrome c synthesis | *ccsA* |
|  | Photosystem biogenesis factor 1 | *pbfl* |
|  | Subunit of rubisco | *rbcL* |
| Transcription and translation | Large subunits of ribosome | *rpl2*(x2), rpl14, rpl16, rpl20, rpl22, rpl23(x2), rpl32, rpl33, rpl36* |
|  | Small subunits of ribosomal proteins | *rps2, rps3, rps4, rps7(x2), rps8, rps11, rps12*(x2), rps14, rps15, rps16*, rps18, rps19, rps19-fragment* |
|  | DNA dependent RNA polymerase | *rpoA, rpoB, rpoC1*, rpoC2* |
|  | Translational initiation factor | *infA* |
|  | rRNA genes | *rrn4.5(x2), rrn5(x2), rrn16(x2), rrn23(x2)* |
|  | tRNA genes | *trnA-UGC*(x2), trnC-GCA, trnD-GUC, trnF-GAA, trnfM-CAU, trnG-UCC, trnH-GUG, trnI-CAU(x2), trnI-GAU*(x2), trnK-UUU*, trnL-CAA(x2), trnL-UAA*, trnL-UAG, trnM-CAU, trnN-GUU(x2), trnP-UGG, trnQ-UUG, trnR-ACG(x2), trnR-UCU, trnS-GCU(x2), trnS-UGA, trnT-GGU, trnT-UGU, trnV-GAC(x2), trnV-UAC*, trnW-CCA, trnY-GUA* |
| Other genes | Maturase K | *matK* |
|  | Envelope membrane protein | *cemA* |
|  | Acetyl-CoA carboxylase | *accD* |

**Supplementary Table3.** List of species and their chloroplast genome accession numbers in NCBI.

| Species | Accession number |
| --- | --- |
| *Chrysanthemum × morifolium* | MH165289.1 |
| *Chrysanthemum × morifolium* var. ‘Anhuishiliuye’ | MT976165.1 |
| *Chrysanthemum × morifolium* var. ‘Hanbaiju’ | MT919681.1 |
| *Chrysanthemum × morifolium* var. ‘wucailongzhua’ | MG873555.1 |
| *Chrysanthemum vestitum* | NC_057203.1 |
| *Chrysanthemum lavandulifolium* | NC_057202.1 |
| *Chrysanthemum indicum* | MH165290.1 |
| *Chrysanthemum przewalskii* | NC_079656.1 |
| *Chrysanthemum dichroum* | NC_064344.1 |
| *Chrysanthemum chanetii* | NC_064343.1 |
| *Chrysanthemum boreale* | NC_037388.1 |
| *Chrysanthemum lucidum* | NC_040920.1 |
| *Chrysanthemum zawadskii* | MK801118.1 |
| *Chrysanthemum glabriusculum* | MN394397.1 |
| *Chrysanthemum oreastrum* | MN385685.1 |
| *Chrysanthemum mongolicum* | MN381811.1 |
| *Chrysanthemum argyrophyllum* | MN460367.1 |
| *Chrysanthemum rhombifolium* | MN233363.1 |
| *Chrysanthemum potentilloides* | MN233362.1 |
| *Chrysanthemum hypargyrum* | MN227074.1 |
| *Helianthus annuus* | NC_007977.1 |
