# Supplementary material for: The complete chloroplast genome sequence of a cultivar of Chrysanthemum, Chrysanthemum morifolium var. ‘Jinsihuang’ (Asteraceae)
Source: Mitochondrial DNA B Resour. 2026 Feb 1;11(3):345–9. doi: 10.1080/23802359.2026.2621429 (PMC12865824; doi:10.1080/23802359.2026.2621429)
Supplement: Clean Version.docx [file TMDN_A_2621429_SM6548.docx]

**The Complete Chloroplast Genome Sequence of** **a cultivar of *Chrysanthemum*, *Chrysanthemum*** ***morifolium* var. 'Jinsihuang' (Asteraceae)**

Wenying Meng^1^, Qimao Wang^2^, Yonghao Wang^2^, Feng Wen^2,*^, Xiaozhu Wu^3,*^

^1^ School of Life Sciences, Anhui University, 230601, Hefei, China

^2^ Anhui Chuju Planting and Deep Processing Engineering Research Center, School of Biological Science and Food Engineering, Chuzhou University, 239000, Chuzhou, China.

^3^ College of Resources and Environment, Jiujiang University, 332000, Jiujiang, China

* Corresponding author: Feng Wen and Xiaozhu Wu

Email address:[wenfeng332@126.com](mailto:wenfeng332@126.com)

xz_wu1983@163.com

Jiulong Road, Economic and Technological Development Zone No.111, Hefei City, Anhui Province, 230601, China

**Abstract**

*Chrysanthemum morifolium* var. 'Jinsihuang' is an ornamental *Chrysanthemum* species with significant medicinal and edible value. In this study, we sequenced, assembled, and annotated the complete chloroplast (cp) genome of *C. morifolium* var. 'Jinsihuang'. The chloroplast genome is 151,060 bp in length, exhibiting a typical quadripartite structure consisting of a large single-copy (LSC) region (82,858 bp), a small single-copy (SSC) region (18,294 bp), and a pair of inverted repeat (IR) regions (24,954 bp each). Phylogenetic analysis revealed that *C. morifolium* var. 'Jinsihuang' is most closely related to *Chrysanthemum × morifolium*. This study provides valuable genomic resources for understanding the phylogenetic relationships and genetic diversity within *C. morifolium* var. 'Jinsihuang', contributing to future research on its evolution and conservation.

**Keywords**

Chloroplast genome; *Chrysanthemum morifolium* var. 'Jinsihuang'; phylogenetic analyses

**1 Introduction**

*Chrysanthemum* *morifolium* var. 'Jinsihuang' (Asteraceae), a perennial herb, is a hybrid variety derived from traditional medicinal and ornamental chrysanthemums (Figure 1). In recent years, it has gained wide popularity among consumers and been exported globally due to its dual value for ornamental and edible purposes. Rich in flavonoids, this cultivar exhibits diverse biological activities (antioxidant, anti-inflammatory, antibacterial, and antiviral) ^[1-2]^ and has traditionally been used in Chinese medicine to treat ailments including headaches, eye fatigue, hypertension, and various cardiovascular diseases ^[3]^. However, molecular-level studies on *C. morifolium* var. 'Jinsihuang' remain limited, and its genetic resources are scarce. In this study, we successfully assembled its complete chloroplast genome and elucidated its phylogenetic position within the *Chrysanthemum* genus. By reporting the first complete plastome of this cultivar, we aim to provide foundational molecular data that will facilitate future comparative genomic, genetic, and taxonomic studies within both the *Chrysanthemum* genus and the Asteraceae family.


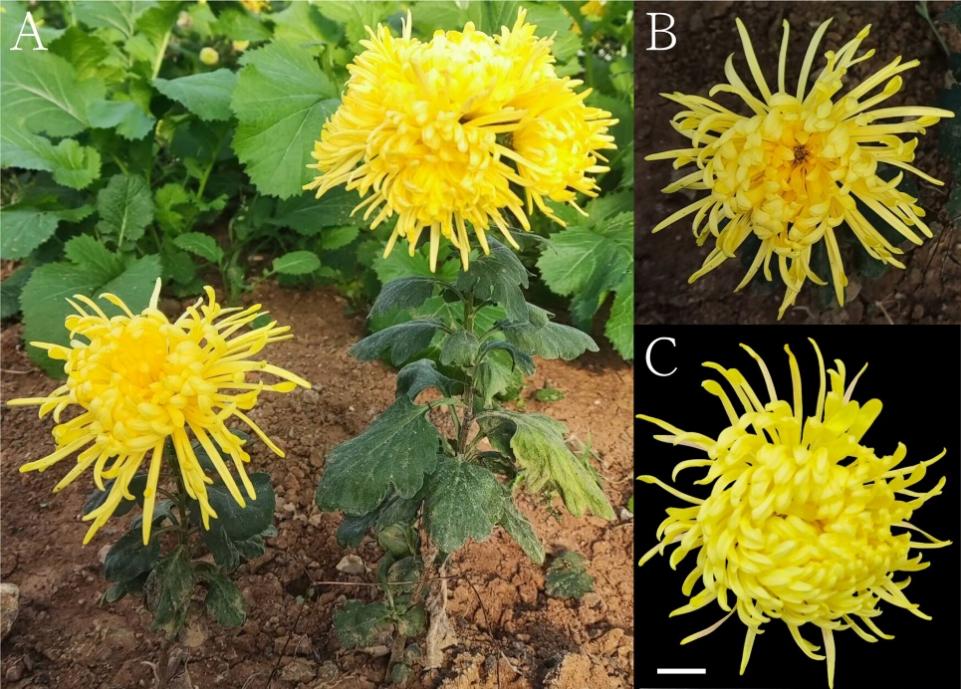


**Figure 1.** The morphological characteristics of *C. morifolium* var. 'Jinsihuang'. (A) Planting site and blooming whole plant. (B) Blooming flowers. (C) Size of the flowers. The scale bar represents 2 cm. Photographs were taken by Wenying Meng at Chuzhou University (32°41'03"N, 118°36'51"E), and a voucher specimen (CZU2023001) is deposited in its Herbarium (CZU), Chuzhou, Anhui, China.

**2** **Materials and Methods**

Fresh leaves of *C. morifolium* var. 'Jinsihuang' were collected from the greenhouse of the Planting and Deep Processing Engineering Research Center, Chuzhou University, Anhui Province, China (32°41'03"N, 118°36'51"E). A voucher specimen (CZU2023001) is deposited in the Herbarium of the School of Biological and Food Engineering, Chuzhou University under the custody of Dr. Feng Wen (email: wenfeng332@126.com). Genomic DNA was extracted from fresh leaves with a modified CTAB method ^[4]^. To mitigate polyphenol and flavonoid interference, PVP-40 concentration to 3% and β-mercaptoethanol to 2% were added. Subsequently, sequencing libraries were constructed following the guidelines provided by the MGIEasy Universal DNA Library Preparation Kit. Afterwards, the 150 bp fragments were sequenced from both ends by utilizing the Illumina HiSeq 2500 platform at Genepioneer Biotechnologies Inc. The raw sequencing data were filtered and trimmed with the help of fastp (version 0.23.4) under its default settings ^[5]^. Altogether, 19.24 million high-quality clean reads were generated, resulting in approximately 5.77 Gb of data. The complete chloroplast genome was assembled using GetOrganelle (version 1.7.7.0). Only top 11408361 pair of reads were used for analysis, and 932053 pair of reads (approximately 8.17% of total used clean reads) were used to assemble the chloroplast genome. The subsequent annotation performed through the GeSeq web-based annotation platform for comprehensive genomic characterization ^[6-7]^. Annotation accuracy was verified using BLAST ([https://blast.ncbi.nlm.nih.gov/Blast.cgi](https://blast.ncbi.nlm.nih.gov/Blast.cgi)) and Geneious (version 8.1.3) ^[8]^. A circular genome map was generated with OGDRAW ^[9]^ using *Arabidopsis thaliana* (NC_000932) as a reference. The start and stop codons were manually corrected based on BLAST results, while cis-spliced and trans-spliced gene profiles were constructed by utilizing CPGview ([http://www.1kmpg.cn/cpgview/)](http://www.1kmpg.cn/cpgview/)%5b10)^[[10](http://www.1kmpg.cn/cpgview/)%5b10)]^. The boundaries of the LSC, SSC, and IR regions were compared using CPJSDraw^[11]^. To evaluate the phylogenetic relationships, chloroplast genomes of 17 *Chrysanthemum* species were retrieved from the NCBI and aligned using ClustalW^[12]^. All sequences from the alignment were used for tree construction without trimming. A maximum likelihood (ML) tree was constructed with MEGA 11 using the GTR+G model (selected as best-fit via AIC) and 1000 bootstrap replicates, with *Helianthus annuus* as the outgroup ^[13]^.

**3 Results**

The complete chloroplast genome of *C. morifolium* var. 'Jinsihuang' was 151,060 bp in length, with mean, maximum, and minimum sequencing depths of 818.64×, 1051×, and 183×, respectively (Supplementary Figure 1). The genome structure comprises a large single-copy (LSC) region with a length of 82,858 bp, a small single-copy (SSC) region measuring 18,294 bp, and two inverted repeat (IR) regions, each being 24,954 bp long, displaying a typical quadripartite structure found in many angiosperms (Figure 2) ^[14]^. The annotated chloroplast genome sequence has been submitted to GenBank with the accession number PQ759883.

The overall GC content of the chloroplast genome amounts to 37.5%, with region-specific values being 35.5% in the LSC region, 30.8% in the SSC region, and 43.1% in the IR regions (Supplementary Table1). The genome contains a total of 130 predicted gene loci. The annotation comprises 87 protein-coding genes (PCGs), 35 tRNA genes, and 8 rRNA genes, with two additional pseudogenes identified. In total, 17 of these genes are duplicated, including *ndhB, rpl2, rpl23, rps7, rps12, rrn4.5, rrn5, rrn16, rrn23, trnA-UGC, trnI-CAU, trnI-GAU, trnL-CAA, trnN-GUU, trnR-ACG, trnS-GCU,* and *trnV-GAC* (Supplementary Table2). Six PCGs (*atpF, rpl2, rpoC1, rps16, ndhA, and ndhB*) contain a single intron, while two PCGs (*pafI, clpP*) have two introns. Notably, the *ndhB* and *rpl2* genes each feature a single duplication (Supplementary Figure 2), while the *rps12* gene undergoes trans-splicing and comprises three distinct exons (Supplementary Figure 3). Although, the chloroplast genome of *C. morifolium* var. 'Jinsihuang' shows significant similarities to other species, differences exist at the junctions of the LSC-IRa (LA) and SSC-IRb (SB) regions compared with specified cultivars and other congeneric *Chrysanthemum* species (Supplementary Figure 4). These structural similarities and differences may indicate the evolutionary connections and relationships among them.


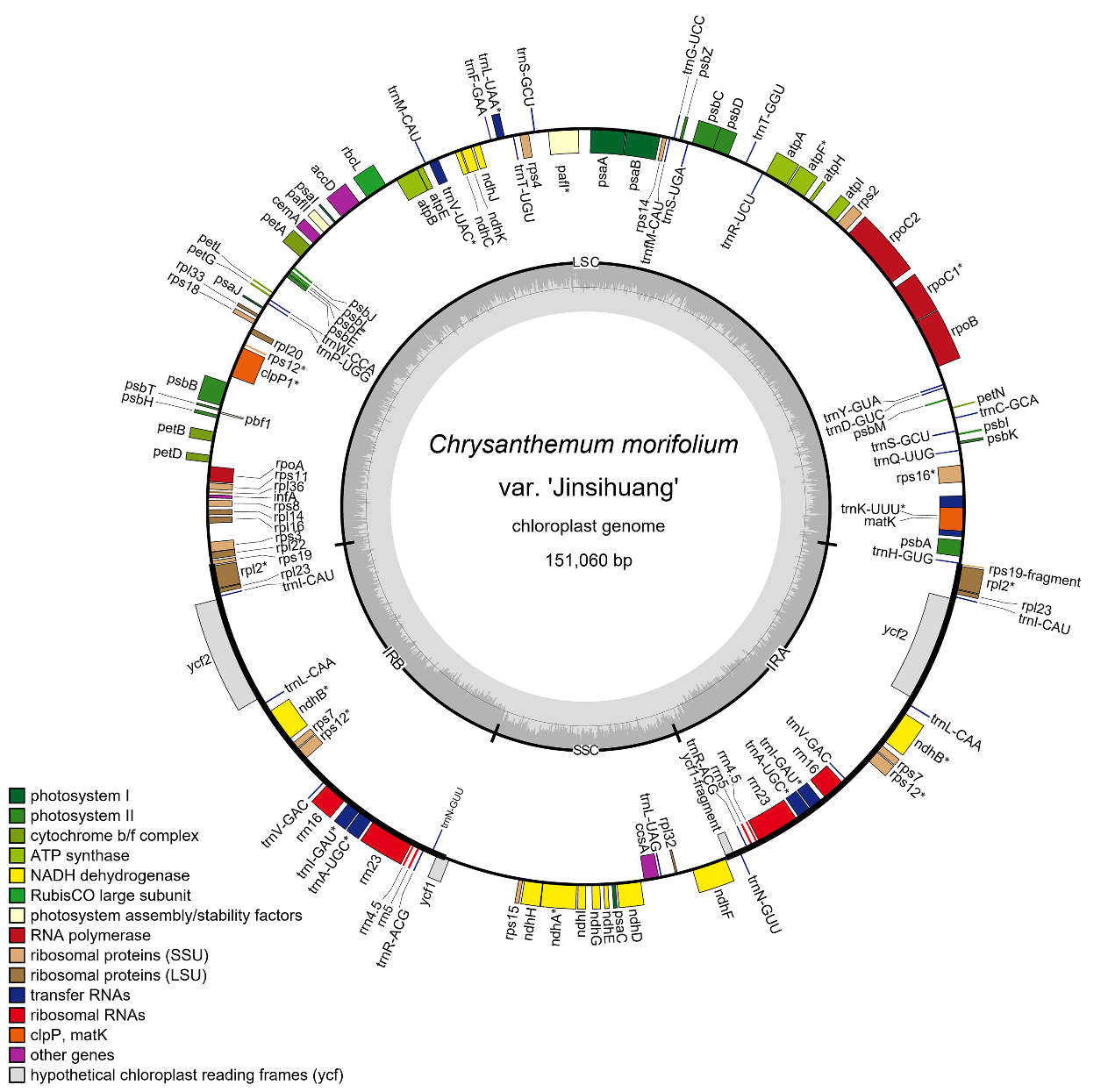


**Figure 2.** Chloroplast genome mapping of *C. morifolium* var. 'Jinsihuang'. The inner circle is a pair of IR regions, LSC and SSC regions. Forward-coding genes are located on the outer side of the circle, while reverse-coding genes are located on the inner side of the circle. The inner gray area circles indicate the GC content. At the bottom, genes with different functional groups are indicated by various colors.

The phylogenetic tree demonstrated that *C.* *morifolium* var. 'Jinsihuang' has the closest genetic relationship with *Chrysanthemum × morifolium* (Figure 3), accompanied by a high bootstrap support value of 100. This tree provides valuable insights into the phylogenetic relationships among *Chrysanthemum* species, highlighting genetic differentiation and establishing a solid foundation for further research into the evolutionary and genetic dynamics of these species.


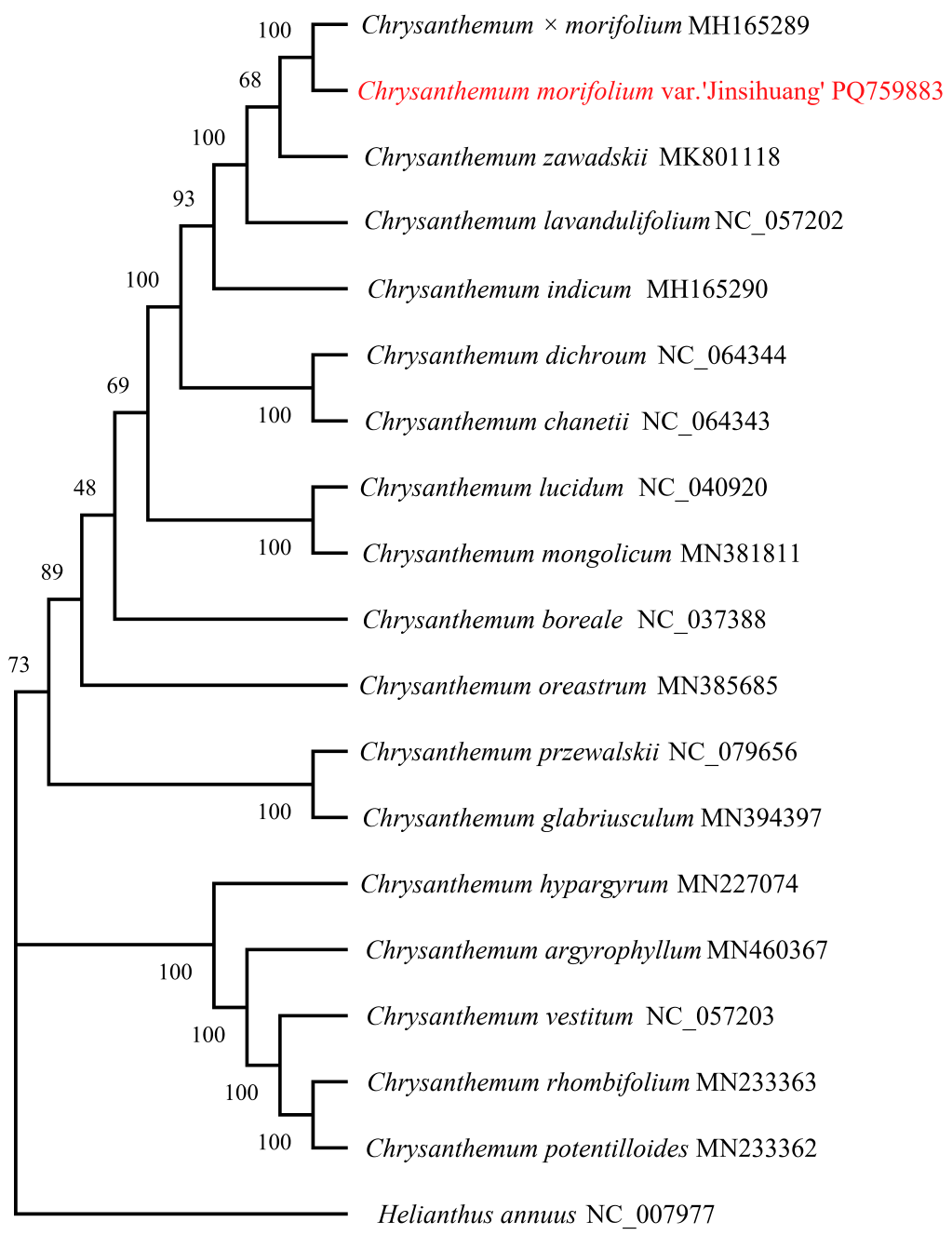


**Figure 3.** Construction of the ML tree based on the chloroplast genomes of 19 species using MEGA 11 and 1000 bootstrap replicates; bootstrap support values are labeled at each branch node. The following sequences were used: MH165289 ^[15]^, PQ759883, NC_057203, NC_057202 ^[15]^, MH165290 ^[16]^, NC_079656, NC_064344, NC_064343, NC_037388 ^[17]^, NC_040920 ^[18]^, MK801118 ^[19]^, MN394397, MN385685, MN381811, MN460367, MN233363, MN233362, MN227074, and NC_007977.

**4 Discussion and Conclusion**

Chloroplasts are vital for sustaining life on Earth, and studying chloroplast genomes offers invaluable insights into the interplay between chloroplast DNA structure and species evolution ^[20]^. In the present study, structural analysis of the *C. morifolium* var. 'Jinsihuang' chloroplast genome revealed two key observations regarding inverted repeat (IR) regions: first, its IR region boundaries exhibit significant similarity to those of other *Chrysanthemum* species, reflecting the conserved nature of IR structures in maintaining cpDNA stability; second, distinct variations were detected at the LSC-IRa (LA) and SSC-IRb (SB) junctions, specifically a shorter *ycf1* gene and a truncated pseudogenized *rps19*, when compared with specified *C. morifolium* cultivars as well as other congeneric *Chrysanthemum* species (Supplementary Figure 4). These junction-specific differences are likely evolutionary signatures, as IR-SC boundaries are known to be dynamic hotspots for cpDNA rearrangement and pseudogenization ^[21-22]^.

Beyond boundary features, the chloroplast genome of 'Jinsihuang' encodes 130 genes, with 17 genes present in duplicate. Gene duplication in IR regions is thought to enhance cpDNA copy number stability and provide functional redundancy, which may buffer against deleterious mutations. Meanwhile, the identification of two pseudogenes in its chloroplast genome provides a unique perspective on genomic evolutionary dynamics ^[22]^. Specifically, the *ycf1*-fragment pseudogene may influence the regulation of chloroplast gene expression, potentially affecting chloroplast development and function. Additionally, the *rps19*-fragment pseudogene could impact chloroplast protein synthesis. The failure to produce functional ribosomal small subunit protein S19 may disrupt the normal assembly of chloroplast ribosomes, highlighting the importance of these genetic elements in chloroplast biology.

Phylogenetic analyses based on chloroplast genome sequences reveal that *C. morifolium* var. 'Jinsihuang' is most closely related to *Chrysanthemum × morifolium*. This close genetic relationship suggests that these species may have faced similar evolutionary selection pressures, leading to shared genetic variations ^[23]^. Furthermore, the unique LA and SB junction differences between *C. morifolium* var. 'Jinsihuang' and other *Chrysanthemum* species offer essential clues for investigating the mechanisms underlying species differentiation within the genus—for instance, whether such structural variations contribute to phenotypic divergence or ecological adaptation.

In summary, our study not only enhances the genomic information of *C. morifolium* var. 'Jinsihuang', but also provides a foundation for understanding genetic diversity, evolution, and phylogenetic relationships within the *Chrysanthemum* genus.

**5 Disclosure statement**

The coauthors do not have any conflict of interest to declare. The authors alone are responsible for the content and composition of the paper.

**6 Ethical approval**

The material involved in the article does not involve ethical conflicts. This species is neither endangered on the CITES catalogue nor collected from a natural reserve, so it did not need specific permissions or licenses. *C. morifolium* var. 'Jinsihuang' is not endangered plant and was collected in accordance with laws and regulations.

**7 Author contributions**

Wenying Meng analyzed and interpreted the genome data and contributed to manuscript writing. Qimao Wang was responsible for sample collection and data acquisition. Yonghao Wang conducted the main experiments and validated the results. Feng Wen and Xiaozhu Wu supervised the study, provided conceptual guidance, and critically reviewed the manuscript. All authors participated in writing and revising the manuscript and agreed to take responsibility for all aspects of the work.

**8 Funding**

This work was supported by the Natural Science Foundation of Anhui Province [2508085MC067], Natural Science Foundation of the Higher Education Institutions of Anhui Province [2023AH051621], Research start-up fund of Chuzhou University [2023qd40], and Natural Science Foundation of Jiangxi Province [20202BABL203045].

**9 Data availability statement**

The genome sequence data that support the findings of this study are openly available in GenBank of NCBI (https://www.ncbi.nlm.nih.gov/) under the accession number PQ759883. The associated BioProject, SRA, and Bio-Sample numbers are PRJNA1214688, SRR33442979, and SAMN46381781, respectively.

**10 References**

[1] Liu J Q, Shen Q Q, Liu J S, et al. studies on the chemical constituents from chrysanthemum morifolium ramat[J]. China journal of Chinese materia medica, 2001;26(8): 547-548.

[2] Kim, J. H., & Kim, Y. S. Anti-inflammatory and analgesic effects of the ethanol extract of Chrysanthemum indicum. Journal of Ethnopharmacology,. Journal of Ethnopharmacology, 2011.137(1), 566-572.

[3] Hadizadeh H , Samiei L, Shakeri A. Chrysanthemum, an ornamental genus with considerable medicinal value: a comprehensive review. South African Journal of Botany, 2022;144:23-43.

[4] Doyle JJ, Doyle JL. A rapid DNA isolation procedure for small quantities of fresh leaf tissue. Phytochem Bull. 1987;19:11-15.

[5] Chen S, Zhou Y, Chen Y, Gu J. fastp: an ultra-fast all-in-one FASTQ preprocessor. Bioinformatics. 2018;34(17):i884-i890.

[6] Jin JJ, Yu WB, Yang JB, Song Y, dePamphilis CW, Yi TS, Li DZ. GetOrganelle: a fast and versatile toolkit for accurate de novo assembly of organelle genomes. Genome Biol. 2020;21, 241.

[7] Tillich M, Lehwark P, Pellizzer T, Ulbricht-Jones ES, Fischer A, Bock R, Greiner S. 2017. GeSeq-versatile and accurate annotation of organelle genomes. Nucleic Acids Res. 45(W1):W6-W11.

[8] Kearse M, Moir R, Wilson A, et al. Geneious Basic: an integrated and extendable desktop software platform for the organization and analysis of sequence data. Bioinformatics. 2012;28(12):1647-1649.

[9] Greiner S, Lehwark P, Bock R. OrganellarGenomeDRAW (OGDRAW) version 1.3.1: expanded toolkit for the graphical visualization of organellar genomes . Nucleic Acids Res. 2019;47(W1):W59-W64.

[10] Liu S, Ni Y, Li J, Zhang X, Yang H, Chen H, Liu C. CPGView: a package for visualizing detailed chloroplast genome structures. Mol Ecol Resour. 2023;23(3):694-704.

[11] Li H, Guo Q, Xu L, Gao H, Liu L, Zhou X. CPJSdraw: analysis and visualization of junction sites of chloroplast genomes. PeerJ. 2023;11:e15326.

[12] Thompson, J. D., Higgins, D. G., & Gibson, T. J. CLUSTAL W: improving the sensitivity of progressive multiple sequence alignment through sequence weighting, position-specific gap penalties and weight matrix choice. Nucleic acids research, 1994;22(22):4673-4680.

[13] Tamura K, Stecher G, Kumar S. MEGA11: molecular evolutionary genetics analysis version 11. Mol Biol Evol. 2021;38(7):3022-3027.

[14] Wicke S, Schneeweiss GM, Depamphilis CW, Müller KF, Quandt D. The evolution of the plastid chromosome in land plants: gene content, gene order, gene function. Plant Mol Biol. 2011;76(3–5):273–297.

[15] Ma YP, Zhao L, Zhang WJ, Zhang YH, Xing X, Duan XX, Hu J, Harris AJ, Liu PL, Dai SL, et al. Origins of cultivars of Chrysanthemum-Evidence from the chloroplast genome and nuclear LFY gene. J of Sytematics Evolution. 2020;58(6):925-944.

[16] Xia, Y., Hu, Z., Li, X., Wang, P., Zhang, X., Li, Q., & Lu, C. The complete chloroplast genome sequence of Chrysanthemum indicum. mitochondrial DNA. part A, DNA mapping, sequencing, and analysis, 2016;27(6):4668-4669.

[17] Won SY, Jung JA, Kim JS. The complete chloroplast genome of Chrysanthemum boreale (Asteraceae). Mitochondrial DNA B Resour. 2018;3(2):549-550.

[18] Kim, J.S., Lee, W., & Pak, J. H. The complete plastid genome sequence of Chrysanthemum lucidum (Asteraceae): an endemic species of Ulleung Island of Korea. Mitochondrial DNA. part B, Resources. 2018;3(2):476-477.

[19] Baek J, Park S, Lee J, Min J, Park J, Lee GW. The complete chloroplast genome of Chrysanthemum zawadskii Herbich (Asteraceae) isolated in Korea. Mitochondrial DNA B Resour. 2021;6(7):1956-1958.

[20] Daniell, H., Lin, C. S., Yu, M., & Chang, W. J. Chloroplast genomes: diversity, evolution, and applications in genetic engineering. Genome Biology, 2016;17(1):134.

[21] Cosner, M.E., Raubeson, L.A. & Jansen, R.K. Chloroplast DNA rearrangements in Campanulaceae: phylogenetic utility of highly rearranged genomes. BMC Evol Biol. 2004;4:27.

[22] Abdullah, Mehmood F, Heidari P, Rahim A, Ahmed I, Poczai P. Pseudogenization of the chloroplast threonine (trnT-GGU) gene in the sunflower family (Asteraceae). Sci Rep. 2021;11(1):21122.

[23] Duan, Y., Wang, Y., Ding, W. *et al.* Comparative and phylogenetic analysis of the chloroplast genomes of four commonly used medicinal cultivars of *Chrysanthemums morifolium*. BMC Plant Biol , 2024;24,992.
